# 3-{2-[2-(Diphenyl­methyl­ene)hydrazin­yl]thia­zol-4-yl}-2*H*-chromen-2-one

**DOI:** 10.1107/S1600536810023627

**Published:** 2010-06-26

**Authors:** Afsheen Arshad, Hasnah Osman, Kit Lam Chan, Chin Sing Yeap, Hoong-Kun Fun

**Affiliations:** a School of Chemical Sciences, Universiti Sains Malaysia, 11800 USM, Penang, Malaysia; b School of Pharmaceutical Sciences, Universiti Sains Malaysia, 11800 USM, Penang, Malaysia; c X-ray Crystallography Unit, School of Physics, Universiti Sains Malaysia, 11800 USM, Penang, Malaysia

## Abstract

In the title compound, C_25_H_17_N_3_O_2_S, the coumarin ring system is essentially planar with a maximum deviation of 0.019 (2) Å. A weak intra­molecular C—H⋯O hydrogen bond stabilizes the mol­ecular structure, so that the coumarin plane is approximately coplanar with the thia­zole ring, making a dihedral angle of 2.5 (10)°. The two phenyl rings are nearly perpendicular to each other, with a dihedral angle of 81.44 (12)°. In the crystal structure, the mol­ecules are linked into an infinite chain along the *b* axis by inter­molecular C—H⋯O hydrogen bonds. Weak C—H⋯π inter­actions are observed between the chains.

## Related literature

For applications of coumarin derivatives, see: Tassies *et al.* (2002 ▶); Laffitte *et al.* (2002 ▶); Weber *et al.* (1998 ▶); Finn *et al.* (2004 ▶); Kimura *et al.* (1985 ▶). For applications of amino­thia­zoles derivatives, see: Hiremath *et al.* (1992 ▶); Karah *et al.* (1998 ▶); Jayashree *et al.* (2005 ▶). For related structures, see: Arshad, Osman, Chan *et al.* (2010*a* ▶,*b* ▶); Arshad, Osman, Lam *et al.* (2010 ▶). For the stability of the temperature controller used for the data collection, see: Cosier & Glazer (1986 ▶). The syntheses of benzophenone thio­semicarbazone and 3-(ω-bromo­acet­yl)coumarin are described by Lobana *et al.* (2006 ▶) and Siddiqui *et al.* (2009 ▶), respectively.
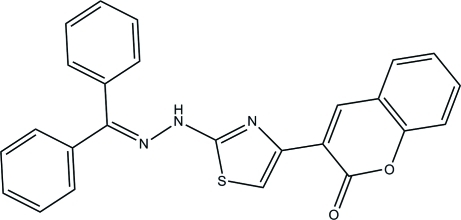


## Experimental

### 

#### Crystal data

C_25_H_17_N_3_O_2_S
*M* *_r_* = 423.48
Monoclinic, 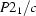

*a* = 13.8705 (18) Å
*b* = 12.9101 (17) Å
*c* = 11.8534 (16) Å
β = 107.563 (2)°
*V* = 2023.6 (5) Å^3^
*Z* = 4
Mo *K*α radiation
μ = 0.19 mm^−1^
*T* = 100 K
0.28 × 0.13 × 0.04 mm

#### Data collection

Bruker APEXII DUO CCD area-detector diffractometer
Absorption correction: multi-scan (*SADABS*; Bruker, 2009 ▶) *T*_min_ = 0.949, *T*_max_ = 0.993
17920 measured reflections
4181 independent reflections
2909 reflections with *I* > 2σ(*I*)
*R*_int_ = 0.067

#### Refinement

*R*[*F* ^2^ > 2σ(*F* ^2^)] = 0.045
*wR*(*F* ^2^) = 0.125
*S* = 1.04
4181 reflections
284 parameters
H atoms treated by a mixture of independent and constrained refinement
Δρ_max_ = 0.24 e Å^−3^
Δρ_min_ = −0.32 e Å^−3^

### 

Data collection: *APEX2* (Bruker, 2009 ▶); cell refinement: *SAINT* (Bruker, 2009 ▶); data reduction: *SAINT*; program(s) used to solve structure: *SHELXTL* (Sheldrick, 2008 ▶); program(s) used to refine structure: *SHELXTL*; molecular graphics: *SHELXTL*; software used to prepare material for publication: *SHELXTL* and *PLATON* (Spek, 2009 ▶).

## Supplementary Material

Crystal structure: contains datablocks global, I. DOI: 10.1107/S1600536810023627/is2564sup1.cif

Structure factors: contains datablocks I. DOI: 10.1107/S1600536810023627/is2564Isup2.hkl

Additional supplementary materials:  crystallographic information; 3D view; checkCIF report

## Figures and Tables

**Table 1:** Hydrogen-bond geometry (Å, °) *Cg*1 and *Cg*2 are the centroids of the C14–C19 and C2–C7 benzene rings, respectively.

*D*—H⋯*A*	*D*—H	H⋯*A*	*D*⋯*A*	*D*—H⋯*A*
C6—H6*A*⋯O1^i^	0.93	2.46	3.377 (3)	168
C11—H11*A*⋯O2	0.93	2.30	2.857 (3)	118
C21—H21*A*⋯*Cg*1^ii^	0.93	2.49	3.387 (3)	162
C24—H24*A*⋯*Cg*2^iii^	0.93	2.78	3.536 (3)	139

Symmetry codes: (i) 
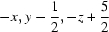
; (ii) 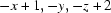; (iii) 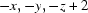.

**Fig. 1.**
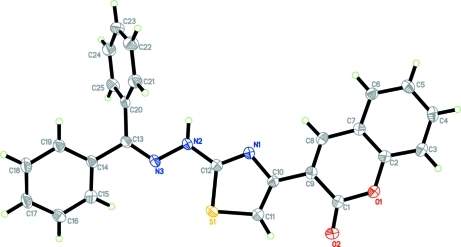
The molecular structure of the title compound with atom labels and 50% probability ellipsoids for non-H atoms.

**Fig. 2.**
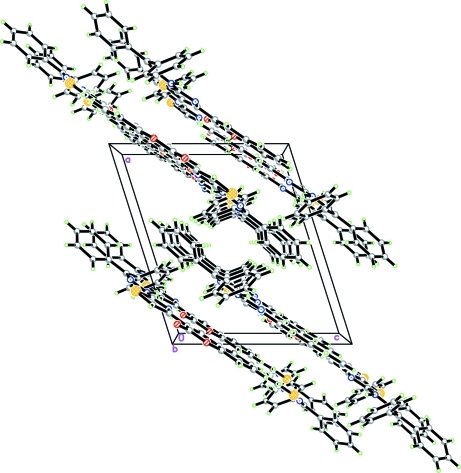
The crystal packing of title compound, viewed down the b axis, showing the molecules are linked into chains along the b axis. Intermolecular hydrogen bonds are shown as dashed lines.

## supplementary crystallographic information

### Comment

Coumarin derivatives having pronounced biological activities are used as anticoagulants (Tassies *et al.*, 2002), antibacterial (Laffitte *et al.*, 2002), cytotoxic (Weber *et al.*, 1998), free radical scavengers (Finn *et al.*, 2004) and enzyme inhibiting (Kimura *et al.*, 1985) agents. Moreover, aminothiazoles derivatives have been reported to exhibit significant antifungal (Hiremath *et al.*, 1992), anti-tuberculosis (Karah *et al.*, 1998) and anti-inflammatory (Jayashree *et al.*, 2005) activities. The title compound is a new coumarinyl thiazolyl hydrazone derivative. We present here its crystal structure.

The geometry parameters of the title compound (Fig. 1) are comparable to those related structures (Arshad, Osman, Chan *et al.*, 2010*a*,*b*; Arshad, Osman, Lam *et al.*, 2010). The coumarin group is essentially planar (O1/C1–C9) with a maximum derivation of 0.019 Å at atom C7. The mean plane is approximately coplanar with the thiazole ring (C10–C11–S1–C12–N1) with a dihedral angle being 2.5 (10)°. The other two benzene rings are nearly perpendicular to each other with a dihedral angle being 81.44 (12)°.

In the crystal structure, the molecules are linked into infinite chains along *b *axis by the intermolecular C6—H6A···O1 hydrogen bonds and stabilized by the weak C—H···π interactions (Fig. 2, Table 1). A weak intramolecular C11—H11A···O2 hydrogen bond stabilizes the molecular structure.

### Experimental

Benzophenone thiosemicarbazone (Lobana *et al.*, 2006) and 3-(ω-bromoacetyl)coumarin (Siddiqui *et al.*, 2009) were synthesized as reported in the literature. A solution of 3-(ω-bromoacetyl)coumarin (2.5 mmol) and benzophenone thiosemicarbazone (2.5 mmol) in chloroform-ethanol (2:1) was refluxed for 1 h. Precipitates formed were filtered and boiled with water containing sodium acetate. The title compound was purified by recrystallization with ethanol-chloroform (1:3) as dark brown feather-like crystals.

### Refinement

H1N2 hydrogen atom was located in a difference Fourier map and was refined freely. The rest of H atoms were positioned geometrically (C–H = 0.93 Å) and refined using a riding model, with *U*_iso_(H) = 1.2*U*_eq_(C).

### Crystal data

**Table:**

C_25_H_17_N_3_O_2_S	*F*(000) = 880
*M**_r_* = 423.48	*D*_x_ = 1.390 Mg m^−^^3^
Monoclinic, *P*2_1_/*c*	Mo *K*α radiation, λ = 0.71073 Å
Hall symbol: -P 2ybc	Cell parameters from 2695 reflections
*a* = 13.8705 (18) Å	θ = 2.2–25.7°
*b* = 12.9101 (17) Å	µ = 0.19 mm^−^^1^
*c* = 11.8534 (16) Å	*T* = 100 K
β = 107.563 (2)°	Plate, brown
*V* = 2023.6 (5) Å^3^	0.28 × 0.13 × 0.04 mm
*Z* = 4	

### Data collection

**Table:**

Bruker APEXII DUO CCD area-detector diffractometer	4181 independent reflections
Radiation source: fine-focus sealed tube	2909 reflections with *I* > 2σ(*I*)
graphite	*R*_int_ = 0.067
φ and ω scans	θ_max_ = 26.5°, θ_min_ = 2.2°
Absorption correction: multi-scan (*SADABS*; Bruker, 2009)	*h* = −17→17
*T*_min_ = 0.949, *T*_max_ = 0.993	*k* = −16→16
17920 measured reflections	*l* = −14→14

### Refinement

**Table:**

Refinement on *F*^2^	Primary atom site location: structure-invariant direct methods
Least-squares matrix: full	Secondary atom site location: difference Fourier map
*R*[*F*^2^ > 2σ(*F*^2^)] = 0.045	Hydrogen site location: inferred from neighbouring sites
*wR*(*F*^2^) = 0.125	H atoms treated by a mixture of independent and constrained refinement
*S* = 1.04	*w* = 1/[σ^2^(*F*_o_^2^) + (0.0488*P*)^2^ + 1.1918*P*] where *P* = (*F*_o_^2^ + 2*F*_c_^2^)/3
4181 reflections	(Δ/σ)_max_ < 0.001
284 parameters	Δρ_max_ = 0.24 e Å^−^^3^
0 restraints	Δρ_min_ = −0.32 e Å^−^^3^

### Special details

**Table:**

Experimental. The crystal was placed in the cold stream of an Oxford Cryosystems Cobra open-flow nitrogen cryostat (Cosier & Glazer, 1986) operating at 100.0 (1) K.
Geometry. All e.s.d.'s (except the e.s.d. in the dihedral angle between two l.s. planes) are estimated using the full covariance matrix. The cell e.s.d.'s are taken into account individually in the estimation of e.s.d.'s in distances, angles and torsion angles; correlations between e.s.d.'s in cell parameters are only used when they are defined by crystal symmetry. An approximate (isotropic) treatment of cell e.s.d.'s is used for estimating e.s.d.'s involving l.s. planes.
Refinement. Refinement of *F*^2^ against ALL reflections. The weighted *R*-factor *wR* and goodness of fit *S* are based on *F*^2^, conventional *R*-factors *R* are based on *F*, with *F* set to zero for negative *F*^2^. The threshold expression of *F*^2^ > σ(*F*^2^) is used only for calculating *R*-factors(gt) *etc*. and is not relevant to the choice of reflections for refinement. *R*-factors based on *F*^2^ are statistically about twice as large as those based on *F*, and *R*- factors based on ALL data will be even larger.

### Fractional atomic coordinates and isotropic or equivalent isotropic displacement parameters (Å^2^)

**Table:**

	*x*	*y*	*z*	*U*_iso_*/*U*_eq_	
S1	0.27268 (5)	0.29137 (5)	0.91250 (6)	0.02688 (18)	
O1	0.03690 (14)	0.49303 (13)	1.21227 (16)	0.0322 (5)	
O2	0.12420 (15)	0.53063 (13)	1.09019 (17)	0.0375 (5)	
N1	0.18366 (15)	0.20621 (15)	1.05262 (17)	0.0216 (4)	
N2	0.25985 (16)	0.08768 (16)	0.95524 (19)	0.0257 (5)	
N3	0.32315 (15)	0.07961 (15)	0.88597 (17)	0.0230 (5)	
C1	0.09314 (19)	0.46244 (19)	1.1393 (2)	0.0267 (6)	
C2	−0.00360 (19)	0.42446 (19)	1.2744 (2)	0.0265 (6)	
C3	−0.0574 (2)	0.4642 (2)	1.3458 (3)	0.0377 (7)	
H3A	−0.0672	0.5352	1.3499	0.045*	
C4	−0.0961 (2)	0.3960 (2)	1.4110 (3)	0.0355 (7)	
H4A	−0.1313	0.4218	1.4604	0.043*	
C5	−0.08363 (19)	0.2904 (2)	1.4042 (2)	0.0274 (6)	
H5A	−0.1104	0.2456	1.4485	0.033*	
C6	−0.03123 (17)	0.25172 (19)	1.3315 (2)	0.0225 (5)	
H6A	−0.0231	0.1805	1.3266	0.027*	
C7	0.00990 (17)	0.31838 (18)	1.2649 (2)	0.0209 (5)	
C8	0.06701 (18)	0.28439 (18)	1.1895 (2)	0.0213 (5)	
H8A	0.0760	0.2137	1.1817	0.026*	
C9	0.10832 (17)	0.35054 (18)	1.1294 (2)	0.0212 (5)	
C10	0.16787 (18)	0.31334 (18)	1.0546 (2)	0.0209 (5)	
C11	0.20939 (19)	0.37041 (19)	0.9838 (2)	0.0268 (6)	
H11A	0.2042	0.4420	0.9753	0.032*	
C12	0.23598 (18)	0.18608 (18)	0.9809 (2)	0.0225 (5)	
C13	0.35407 (18)	−0.01140 (18)	0.8675 (2)	0.0210 (5)	
C14	0.42271 (18)	−0.01483 (18)	0.7927 (2)	0.0213 (5)	
C15	0.42459 (18)	0.06486 (19)	0.7143 (2)	0.0232 (5)	
H15A	0.3784	0.1189	0.7037	0.028*	
C16	0.49430 (19)	0.0646 (2)	0.6520 (2)	0.0261 (6)	
H16A	0.4949	0.1184	0.6000	0.031*	
C17	0.5631 (2)	−0.0155 (2)	0.6670 (2)	0.0288 (6)	
H17A	0.6097	−0.0156	0.6247	0.035*	
C18	0.56302 (19)	−0.0957 (2)	0.7446 (2)	0.0284 (6)	
H18A	0.6098	−0.1492	0.7549	0.034*	
C19	0.49287 (18)	−0.09584 (19)	0.8069 (2)	0.0237 (5)	
H19A	0.4923	−0.1500	0.8585	0.028*	
C20	0.33051 (17)	−0.11039 (18)	0.9191 (2)	0.0210 (5)	
C21	0.36721 (19)	−0.12891 (19)	1.0402 (2)	0.0268 (6)	
H21A	0.4018	−0.0770	1.0907	0.032*	
C22	0.3526 (2)	−0.2240 (2)	1.0859 (2)	0.0288 (6)	
H22A	0.3778	−0.2360	1.1669	0.035*	
C23	0.30028 (19)	−0.3019 (2)	1.0112 (2)	0.0273 (6)	
H23A	0.2916	−0.3664	1.0417	0.033*	
C24	0.26132 (19)	−0.28293 (19)	0.8913 (2)	0.0282 (6)	
H24A	0.2250	−0.3342	0.8411	0.034*	
C25	0.27632 (18)	−0.18767 (19)	0.8457 (2)	0.0238 (5)	
H25A	0.2498	−0.1753	0.7649	0.029*	
H1N2	0.2549 (19)	0.034 (2)	1.003 (2)	0.027 (7)*	

### Atomic displacement parameters (Å^2^)

**Table:**

	*U*^11^	*U*^22^	*U*^33^	*U*^12^	*U*^13^	*U*^23^
S1	0.0327 (4)	0.0239 (3)	0.0311 (4)	−0.0054 (3)	0.0203 (3)	−0.0020 (3)
O1	0.0445 (11)	0.0185 (9)	0.0433 (11)	0.0005 (8)	0.0277 (10)	−0.0013 (8)
O2	0.0531 (13)	0.0196 (9)	0.0524 (12)	−0.0013 (9)	0.0350 (11)	0.0020 (9)
N1	0.0227 (10)	0.0206 (10)	0.0244 (11)	0.0010 (9)	0.0116 (9)	−0.0006 (9)
N2	0.0305 (12)	0.0211 (11)	0.0344 (12)	0.0012 (9)	0.0230 (11)	0.0009 (10)
N3	0.0245 (11)	0.0237 (11)	0.0260 (11)	−0.0008 (9)	0.0153 (10)	−0.0015 (9)
C1	0.0319 (14)	0.0208 (13)	0.0312 (14)	0.0010 (11)	0.0153 (13)	−0.0020 (11)
C2	0.0302 (14)	0.0219 (13)	0.0313 (14)	−0.0006 (11)	0.0153 (12)	0.0014 (11)
C3	0.0533 (19)	0.0200 (13)	0.0517 (18)	0.0034 (13)	0.0336 (16)	−0.0029 (13)
C4	0.0405 (16)	0.0312 (15)	0.0456 (17)	0.0047 (12)	0.0292 (15)	−0.0048 (13)
C5	0.0262 (13)	0.0299 (14)	0.0304 (14)	−0.0022 (11)	0.0150 (12)	0.0003 (11)
C6	0.0223 (12)	0.0202 (12)	0.0254 (13)	−0.0009 (10)	0.0077 (11)	−0.0005 (10)
C7	0.0182 (12)	0.0224 (13)	0.0211 (12)	−0.0005 (10)	0.0046 (10)	−0.0008 (10)
C8	0.0208 (12)	0.0188 (12)	0.0227 (12)	0.0003 (10)	0.0044 (11)	−0.0027 (10)
C9	0.0184 (12)	0.0222 (13)	0.0220 (12)	−0.0015 (10)	0.0048 (11)	−0.0036 (10)
C10	0.0217 (12)	0.0184 (12)	0.0241 (12)	−0.0017 (10)	0.0091 (11)	−0.0020 (10)
C11	0.0329 (14)	0.0202 (13)	0.0321 (14)	−0.0029 (11)	0.0172 (13)	−0.0030 (11)
C12	0.0224 (13)	0.0215 (13)	0.0262 (13)	−0.0012 (10)	0.0113 (11)	−0.0002 (10)
C13	0.0209 (12)	0.0231 (13)	0.0210 (12)	−0.0017 (10)	0.0094 (11)	−0.0010 (10)
C14	0.0237 (13)	0.0222 (13)	0.0205 (12)	−0.0039 (10)	0.0106 (11)	−0.0051 (10)
C15	0.0230 (13)	0.0269 (13)	0.0182 (12)	0.0006 (11)	0.0040 (11)	−0.0019 (10)
C16	0.0301 (14)	0.0316 (14)	0.0182 (13)	−0.0013 (11)	0.0096 (12)	0.0012 (11)
C17	0.0310 (14)	0.0332 (15)	0.0287 (14)	−0.0007 (12)	0.0189 (13)	−0.0057 (12)
C18	0.0296 (14)	0.0305 (14)	0.0298 (14)	0.0035 (11)	0.0161 (13)	−0.0047 (11)
C19	0.0263 (13)	0.0237 (13)	0.0240 (13)	−0.0030 (10)	0.0119 (12)	−0.0039 (10)
C20	0.0196 (12)	0.0209 (12)	0.0269 (13)	0.0007 (10)	0.0137 (11)	−0.0015 (10)
C21	0.0285 (14)	0.0250 (13)	0.0283 (14)	−0.0027 (11)	0.0106 (12)	−0.0017 (11)
C22	0.0309 (14)	0.0321 (15)	0.0223 (13)	0.0011 (11)	0.0062 (12)	0.0043 (11)
C23	0.0279 (14)	0.0249 (13)	0.0311 (14)	−0.0021 (11)	0.0120 (12)	0.0040 (11)
C24	0.0296 (14)	0.0256 (14)	0.0312 (15)	−0.0059 (11)	0.0119 (12)	−0.0043 (11)
C25	0.0227 (13)	0.0306 (14)	0.0174 (12)	−0.0039 (11)	0.0052 (11)	−0.0025 (10)

### Geometric parameters (Å, °)

**Table:**

S1—C11	1.724 (2)	C10—C11	1.368 (3)
S1—C12	1.736 (2)	C11—H11A	0.9300
O1—C2	1.375 (3)	C13—C14	1.485 (3)
O1—C1	1.386 (3)	C13—C20	1.495 (3)
O2—C1	1.205 (3)	C14—C15	1.392 (3)
N1—C12	1.300 (3)	C14—C19	1.404 (3)
N1—C10	1.402 (3)	C15—C16	1.382 (3)
N2—C12	1.370 (3)	C15—H15A	0.9300
N2—N3	1.375 (2)	C16—C17	1.382 (3)
N2—H1N2	0.91 (3)	C16—H16A	0.9300
N3—C13	1.292 (3)	C17—C18	1.385 (3)
C1—C9	1.470 (3)	C17—H17A	0.9300
C2—C3	1.385 (3)	C18—C19	1.388 (3)
C2—C7	1.391 (3)	C18—H18A	0.9300
C3—C4	1.382 (4)	C19—H19A	0.9300
C3—H3A	0.9300	C20—C25	1.387 (3)
C4—C5	1.380 (4)	C20—C21	1.391 (3)
C4—H4A	0.9300	C21—C22	1.382 (3)
C5—C6	1.378 (3)	C21—H21A	0.9300
C5—H5A	0.9300	C22—C23	1.391 (4)
C6—C7	1.400 (3)	C22—H22A	0.9300
C6—H6A	0.9300	C23—C24	1.382 (4)
C7—C8	1.430 (3)	C23—H23A	0.9300
C8—C9	1.346 (3)	C24—C25	1.384 (3)
C8—H8A	0.9300	C24—H24A	0.9300
C9—C10	1.464 (3)	C25—H25A	0.9300
			
C11—S1—C12	88.29 (11)	N1—C12—S1	116.69 (18)
C2—O1—C1	123.27 (19)	N2—C12—S1	119.88 (16)
C12—N1—C10	109.20 (19)	N3—C13—C14	115.7 (2)
C12—N2—N3	116.31 (19)	N3—C13—C20	125.68 (19)
C12—N2—H1N2	119.7 (16)	C14—C13—C20	118.54 (19)
N3—N2—H1N2	119.8 (16)	C15—C14—C19	118.6 (2)
C13—N3—N2	118.33 (19)	C15—C14—C13	121.4 (2)
O2—C1—O1	116.4 (2)	C19—C14—C13	119.8 (2)
O2—C1—C9	126.8 (2)	C16—C15—C14	120.8 (2)
O1—C1—C9	116.8 (2)	C16—C15—H15A	119.6
O1—C2—C3	118.1 (2)	C14—C15—H15A	119.6
O1—C2—C7	120.3 (2)	C17—C16—C15	120.0 (2)
C3—C2—C7	121.6 (2)	C17—C16—H16A	120.0
C4—C3—C2	118.5 (2)	C15—C16—H16A	120.0
C4—C3—H3A	120.7	C16—C17—C18	120.4 (2)
C2—C3—H3A	120.7	C16—C17—H17A	119.8
C5—C4—C3	121.3 (2)	C18—C17—H17A	119.8
C5—C4—H4A	119.3	C17—C18—C19	119.7 (2)
C3—C4—H4A	119.3	C17—C18—H18A	120.1
C6—C5—C4	119.6 (2)	C19—C18—H18A	120.1
C6—C5—H5A	120.2	C18—C19—C14	120.5 (2)
C4—C5—H5A	120.2	C18—C19—H19A	119.8
C5—C6—C7	120.8 (2)	C14—C19—H19A	119.8
C5—C6—H6A	119.6	C25—C20—C21	119.0 (2)
C7—C6—H6A	119.6	C25—C20—C13	120.1 (2)
C2—C7—C6	118.2 (2)	C21—C20—C13	120.8 (2)
C2—C7—C8	117.8 (2)	C22—C21—C20	120.4 (2)
C6—C7—C8	124.1 (2)	C22—C21—H21A	119.8
C9—C8—C7	122.7 (2)	C20—C21—H21A	119.8
C9—C8—H8A	118.6	C21—C22—C23	120.2 (2)
C7—C8—H8A	118.6	C21—C22—H22A	119.9
C8—C9—C10	121.4 (2)	C23—C22—H22A	119.9
C8—C9—C1	119.2 (2)	C24—C23—C22	119.6 (2)
C10—C9—C1	119.4 (2)	C24—C23—H23A	120.2
C11—C10—N1	115.1 (2)	C22—C23—H23A	120.2
C11—C10—C9	127.9 (2)	C23—C24—C25	120.1 (2)
N1—C10—C9	116.96 (19)	C23—C24—H24A	120.0
C10—C11—S1	110.66 (18)	C25—C24—H24A	120.0
C10—C11—H11A	124.7	C24—C25—C20	120.7 (2)
S1—C11—H11A	124.7	C24—C25—H25A	119.6
N1—C12—N2	123.4 (2)	C20—C25—H25A	119.6
			
C12—N2—N3—C13	174.6 (2)	C10—N1—C12—N2	−176.8 (2)
C2—O1—C1—O2	−179.9 (2)	C10—N1—C12—S1	1.2 (3)
C2—O1—C1—C9	0.3 (4)	N3—N2—C12—N1	−174.4 (2)
C1—O1—C2—C3	179.2 (3)	N3—N2—C12—S1	7.6 (3)
C1—O1—C2—C7	−0.7 (4)	C11—S1—C12—N1	−1.5 (2)
O1—C2—C3—C4	−178.3 (3)	C11—S1—C12—N2	176.6 (2)
C7—C2—C3—C4	1.6 (4)	N2—N3—C13—C14	−179.6 (2)
C2—C3—C4—C5	−1.1 (5)	N2—N3—C13—C20	−2.6 (4)
C3—C4—C5—C6	0.2 (4)	N3—C13—C14—C15	−22.3 (3)
C4—C5—C6—C7	0.3 (4)	C20—C13—C14—C15	160.5 (2)
O1—C2—C7—C6	178.9 (2)	N3—C13—C14—C19	152.8 (2)
C3—C2—C7—C6	−1.0 (4)	C20—C13—C14—C19	−24.5 (3)
O1—C2—C7—C8	0.2 (4)	C19—C14—C15—C16	−0.2 (4)
C3—C2—C7—C8	−179.6 (3)	C13—C14—C15—C16	174.9 (2)
C5—C6—C7—C2	0.0 (4)	C14—C15—C16—C17	0.1 (4)
C5—C6—C7—C8	178.6 (2)	C15—C16—C17—C18	−0.3 (4)
C2—C7—C8—C9	0.7 (4)	C16—C17—C18—C19	0.5 (4)
C6—C7—C8—C9	−177.9 (2)	C17—C18—C19—C14	−0.6 (4)
C7—C8—C9—C10	178.9 (2)	C15—C14—C19—C18	0.5 (4)
C7—C8—C9—C1	−1.1 (4)	C13—C14—C19—C18	−174.7 (2)
O2—C1—C9—C8	−179.1 (3)	N3—C13—C20—C25	117.4 (3)
O1—C1—C9—C8	0.6 (4)	C14—C13—C20—C25	−65.7 (3)
O2—C1—C9—C10	0.9 (4)	N3—C13—C20—C21	−66.3 (3)
O1—C1—C9—C10	−179.4 (2)	C14—C13—C20—C21	110.6 (3)
C12—N1—C10—C11	−0.1 (3)	C25—C20—C21—C22	2.0 (3)
C12—N1—C10—C9	178.9 (2)	C13—C20—C21—C22	−174.3 (2)
C8—C9—C10—C11	176.1 (3)	C20—C21—C22—C23	−0.5 (4)
C1—C9—C10—C11	−3.9 (4)	C21—C22—C23—C24	−1.3 (4)
C8—C9—C10—N1	−2.8 (3)	C22—C23—C24—C25	1.4 (4)
C1—C9—C10—N1	177.2 (2)	C23—C24—C25—C20	0.1 (4)
N1—C10—C11—S1	−1.0 (3)	C21—C20—C25—C24	−1.9 (3)
C9—C10—C11—S1	−179.9 (2)	C13—C20—C25—C24	174.5 (2)
C12—S1—C11—C10	1.3 (2)		

### Hydrogen-bond geometry (Å, °)

**Table:**

Cg1 and Cg2 are the centroids of the C14–C19 and C2–C7 benzene rings, respectively.

**Table:**

*D*—H···*A*	*D*—H	H···*A*	*D*···*A*	*D*—H···*A*
C6—H6A···O1^i^	0.93	2.46	3.377 (3)	168
C11—H11A···O2	0.93	2.30	2.857 (3)	118
C21—H21A···Cg1^ii^	0.93	2.49	3.387 (3)	162
C24—H24A···Cg2^iii^	0.93	2.78	3.536 (3)	139

Symmetry codes: (i) −*x*, *y*−1/2, −*z*+5/2; (ii) −*x*+1, −*y*, −*z*+2; (iii) −*x*, −*y*, −*z*+2.
